# Nighttime aircraft noise impairs endothelial function and increases blood pressure in patients with or at high risk for coronary artery disease

**DOI:** 10.1007/s00392-014-0751-x

**Published:** 2014-08-22

**Authors:** Frank Schmidt, Kristoffer Kolle, Katharina Kreuder, Boris Schnorbus, Philip Wild, Marlene Hechtner, Harald Binder, Tommaso Gori, Thomas Münzel

**Affiliations:** 1 2 Medical Clinic, Cardiology, University Medical Center Mainz, Johannes Gutenberg University, Langenbeckstrasse 1, 55131 Mainz, Germany; 2 Institute for Medical Statistics and Biometrics, Mainz, Germany

**Keywords:** Endothelial function, Coronary artery disease, Night time aircraft noise, Arterial hypertension, Annoyance, Sleeping quality

## Abstract

**Aims:**

Epidemiological studies suggest the existence of a relationship between aircraft noise exposure and increased risk for myocardial infarction and stroke. Patients with established coronary artery disease and endothelial dysfunction are known to have more future cardiovascular events. We therefore tested the effects of nocturnal aircraft noise on endothelial function in patients with or at high risk for coronary artery disease.

**Methods:**

60 Patients (50p 1–3 vessels disease; 10p with a high Framingham Score of 23 %) were exposed in random and blinded order to aircraft noise and no noise conditions. Noise was simulated in the patients’ bedroom and consisted of 60 events during one night. Polygraphy was recorded during study nights, endothelial function (flow-mediated dilation of the brachial artery), questionnaires and blood sampling were performed on the morning after each study night.

**Results:**

The mean sound pressure levels *L*_eq(3)_ measured were 46.9 ± 2.0 dB(A) in the Noise 60 nights and 39.2 ± 3.1 dB(A) in the control nights. Subjective sleep quality was markedly reduced by noise from 5.8 ± 2.0 to 3.7 ± 2.2 (*p* < 0.001). FMD was significantly reduced (from 9.6 ± 4.3 to 7.9 ± 3.7 %; *p* < 0.001) and systolic blood pressure was increased (from 129.5 ± 16.5 to 133.6 ± 17.9 mmHg; *p* = 0.030) by noise. The adverse vascular effects of noise were independent from sleep quality and self-reported noise sensitivity.

**Conclusions:**

Nighttime aircraft noise markedly impairs endothelial function in patients with or at risk for cardiovascular disease. These vascular effects appear to be independent from annoyance and attitude towards noise and may explain in part the cardiovascular side effects of nighttime aircraft noise.

## Introduction

The role of noise as an environmental pollutant affecting health has been increasingly recognized. While acute noise interferes with communication, disturbs sleep and causes annoyance, chronic noise exposure has been demonstrated to be associated with negative health outcomes (for review [1]). Studies demonstrated a significant increase in blood pressure (HYENA) in adults [2] and children (RANCH) [3], an increase in prescriptions of cardiovascular medications [4] as well as an increase in heart disease and stroke (for review [5]).

A recent investigator-blinded field study (The FLIGHT-Study) from our group demonstrated that simulated nighttime aircraft noise leads to endothelial dysfunction, worsening of sleep quality and increased vascular stiffness but no significant changes in blood pressure in young healthy volunteers [6].

In an accompanying editorial Charakido and Deanfield emphasized that when considering the relevance of the findings for long-term clinical outcomes as well as for the causal pathways to cardiovascular disease and complications, that it would be highly important to examine the effects of noise on endothelial function of patients with already established cardiovascular disease [7].

Thus, the FLIGHT-RISK study was set out to test the effect of nocturnal aircraft noise on endothelial function, stress hormone levels, blood pressure and inflammatory markers, sleeping quality, annoyance levels and coagulation markers in patients with established coronary artery disease or at high risk for developing coronary artery disease based on the Framingham score.

## Methods

The study was approved by the local ethics committee. All volunteers signed informed consent. Anti-aircraft noise activists and airport employees were excluded from the study as were persons with high nighttime traffic noise exposure at home as determined by noise maps available from municipal online resources (*L*_A,eq,22-6h_ > 40 dB for aircraft noise and *L*_A,eq,22-6h_ > 45 dB for road and rail traffic noise).

### Study population

Men and women between 30 and 75 years of age with either established cardiovascular disease or a 10y cardiovascular risk of at least 10 % as calculated by the Framingham General CVD risk calculator were enrolled.

Patients had to be in a stable clinical condition without hospitalization or medication change in the preceding four weeks. Patients with NYHA III-IV heart failure, severe aortic stenosis, uncontrolled blood pressure (> 160/100 mmHg) and heart rate > 120 bpm were excluded.

Persons with sleeping disorders (Pittsburgh sleep quality index, PSQI > 10), sleep disordered breathing, hearing loss >30 dB(A) and shift workers were also excluded. All blood pressure agents except for nitrates were allowed, calcium-antagonists had to be stopped 48 h prior to testing.

Patients were mainly recruited via flyers and posters at the clinic and cardiologists offices.

### Study procedures

After initial screening and baseline data collection, subjects were exposed to simulated aircraft noise and no noise conditions in a randomized, cross-over and investigator-blinded fashion. The noise simulation took place in the familiar surroundings of the participants’ own bedrooms, thereby minimizing effects of an artificial laboratory situation.

The aircraft noise consisted of 60 repetitive noise events, which had been recorded near Düsseldorf airport (window tilted open) have been used in our previous study [6]. Silent periods of two different durations were inserted between noise events [6]. During the study nights, polygraphic data were collected with devices (SOMNOwatch, SOMNOmedics, Randersacker, Germany) worn on the participants’ body. Sound pressure levels were continuously recorded in the bedroom with class-2 sound level meters to detect external noises and assure compliance. The noise started after a 39.5 min silent period to facilitate sleep onset. The last noise event was played back after 415 min, each noise event lasting roughly 45 s. After each study night, the participants returned to the study center for flow-mediated dilation (FMD) measurements and blood sample collection.

Study participants were instructed to refrain from the consumption of coffee, tea, alcohol, sleep altering medications and nicotine on the day prior to the study night.

Participants attitude towards air traffic, aircraft noise and airport expansion was assessed with a dedicated questionnaire consisting of 19 items contributing to a total score between 0–64 with higher values denoting a more negative attitude.

In a subset of patients (*n* = 19), citrated whole blood was centrifuged and frozen according to standard protocol for later analysis of coagulation factors.

FMD of the brachial artery was measured at the same time in the early morning by a technician using standardized technique described previously [8].

Blood pressure was measured continuously during the study night with the polygraphy device using the pulse transit time method. Given values are averaged over the 8 h period.

### Statistical analysis

The level of significance for the primary endpoint (FMD) was set to 5 %. The analyses of secondary outcomes were regarded as explorative without adjustment for multiple testing. Differences between baseline characteristics were analyzed using paired *t* tests or paired Wilcoxon tests as appropriate. Linear mixed models were used to analyze differences between noise and control nights. These models were adjusted for gender, age, night sequence, PSQI, overall noise sensitivity (NoiSeQ), sleep related noise sensitivity, attitude towards aircraft noise, and morningness-eveningness questionnaire (MEQ).

An interim analysis was scheduled at 60 patients; the stopping rule was based on the Haybittle-Peto boundary, i.e. it was predefined that the trial should be stopped early for a *p* < 0.001 between visits [9, 10]. Statistical analysis was performed using IBM SPSS Statistics Version 21.

## Results

### Patient characteristics and study variables (Table 1)

60 patients (m:w = 44:16) with a mean age of 61.8 ± 9.2 years were analyzed. The average calculated Framingham risk score was 26 %, (range 3–59 %). 50 patients had an established diagnosis of coronary artery disease (CAD) based on coronary angiograms (Table 1), the remaining ten had a Framingham risk score of 23.4 ± 11.4 %. The study population did not have relevant sleep disorders as determined with the PSQI. According to the MEQ, 25 % of patients were classified as evening types and 30 % as morning types, the rest in the indeterminate range. Further information about the study population is given in Table 2 ***.***

**Table 1:** Baseline characteristics of the study population

Parameter	Total (*n* = 60)
Age (year)	61.8 ± 9.2
Male (*n* %)	44 (73.3)
BMI (kg/m^2^)	27.1 ± 3.7
Framingham score	26.0 ± 14.3
Previous MI (*n* %)	35(58.3)
CAD *n* (%)	50 (83.3)
1-vessel disease	18 (30)
2	17 (28.3)
3	15 (25)
Baseline noise sensitivity, sleep quality index, chronotype	
PSQI	4.4 ± 2.2
NoiSeQ	1.5 ± 0.4
Mequation (14–84)	59.3 ± 9.87
Laboratory values	
LDL (mg/dl)	102.6 ± 30.9
HDL (mg/dl)	49.8 ± 13.6
Triglycerides (mg/dl)	184.73 ± 105.1
CRP(mg/l)	2.5 ± 4.2
Creatinin (md/dl)	0.96 ± 0.20
Hemodynamic values	
Office BP (mmHg)	137/74
Heart rate (bpm)	61.0 ± 7.9
Medication *n* (%)	
ASS or clopidogrel	47 (78.3)
ACE-I/AT-1 antagonists	39 (65.0)
Beta-blockers	41 (68.3)
Statins	37 (61.7)
Diuretics	21 (35)

Data are presented as mean ± SD

*BMI* body mass index, *MI* myocardial infarction, *CAD* coronary artery disease, *CRP* C-reactive protein, *NoiSeQ* dortmund noise sensitivity questionnaire with three greatest noise sensitivity, *MEQ* (Horne-Östberg) morningness-eveningness questionnaire, *PSQI* pittsburg sleep quality index, *BP* blood pressure

**Table 2:** Effects of nighttime noise on the quality of sleep, hemodynamic and neurohormonal parameters and markers for inflammation

	Control	Noise 60	P (mixed model)
L_eq3_ dB(A)	39.2 ± 3.1	46.9 ± 2.0	**<0.001**
Sleep quality	5.8 ± 2.0	3.7 ± 2.2	**<0.001**
PTT mean (ms)	322.3 ± 20.7	323.3 ± 20.4	0.450
PTT min (ms)	273.1 ± 21.1	273.3 ± 21.3	0.963
HR mean	60.7 ± 7.9	61.2 ± 7.9	0.320
HR max	93.1 ± 19.1	93.1 ± 14.3	0.951
BPsys mean (mm Hg)	129.5 ± 16.5	133.6 ± 17.9	**0.030**
BPrise	5.3 ± 7.8	6.4 ± 8.2	0.120
HR_accel	8.9 ± 15.3	13.5 ± 25.5	0.059
Adrenaline (ng/l)	36.8 ± 18.0	38.1 ± 27.6	0.504
Cortisol (µg/l)	11.7 ± 3.4	11.2 ± 3.3	0.219
Neutrophils (%)	60.3 ± 7.8	60.8 ± 8.0	0.585
IL-6 (pg/ml)	4.1 ± 6.3	4.1 ± 7.6	0.697
CRP (mg/l)	2.5 ± 4.2	2.5 ± 4.2	0.959

Data are mean ± SD

Bold values indicate statistically significance at the 5 % level

*L*_*eq3*_ *dB * long-term continuous sound level, *PTT* pulse transit time, *HR* heart rate, *BPsyst* systolic blood pressure, *IL-6* interleukin 6, *CRP* C-reactive protein

There was no evidence for differences in outside temperatures (*p* = 0.411) or humidity (*p* = 0.815 between the study nights). Participants’ blood pressure before the start of noise simulation were 137/74 mmHg (control night) and 136/75 mmHg (noise night; *p* = 0.556).

Averaged sound pressure levels L_eq(3)_ were 46.9 ± 2.0 dB(A) in the noise nights and 39.2 ± 3.1 dB(A) in the control nights.

### Primary outcome

**Fig. 1:**
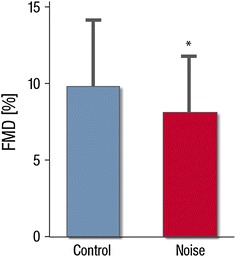
Effects of nighttime noise on flow-mediated dilation (FMD) in patients with or being at risk for coronary artery disease. Data are mean ± SD of 60 patients, *p < 0.001 adjusted for gender, age, night sequence, PSQI, overall noise sensitivity (NoiSeQ), sleep related noise sensitivity, attitude towards aircraft noise, and the results of the Morning Evening Questionnaire

Compared to study nights without noise simulation, FMD of the brachial artery was markedly reduced after nighttime aircraft noise exposure (FMD respectively 9.6 ± 4.3 and 7.9 ± 3.7 %; *p* < 0.001; Fig. 1). Neither baseline vessel diameter (*p* = 0.442) nor velocity time integral (*p* = 0.348) changed significantly between control and noise nights.

The randomization sequence had no impact on the blunting in FMD induced by noise (FMD on the first study night 8.7 ± 4.1 vs. 8.8 ± 4.1 % on the second night; *p* = 0.980).

Noise exposure was associated with impairment in FMD in both subjects at higher risk (Framingham risk score ≥22 %) and those at lower risk.

For the lower risk group, FMD changed from 10.1 ± 4.3 to 8.0 ± 3.2 % (*p* = 0.001) and for the higher risk group FMD was reduced from 9.1 ± 4.4 to 7.8 ± 4.2 % (*p* = 0.023).

**Fig. 2:**
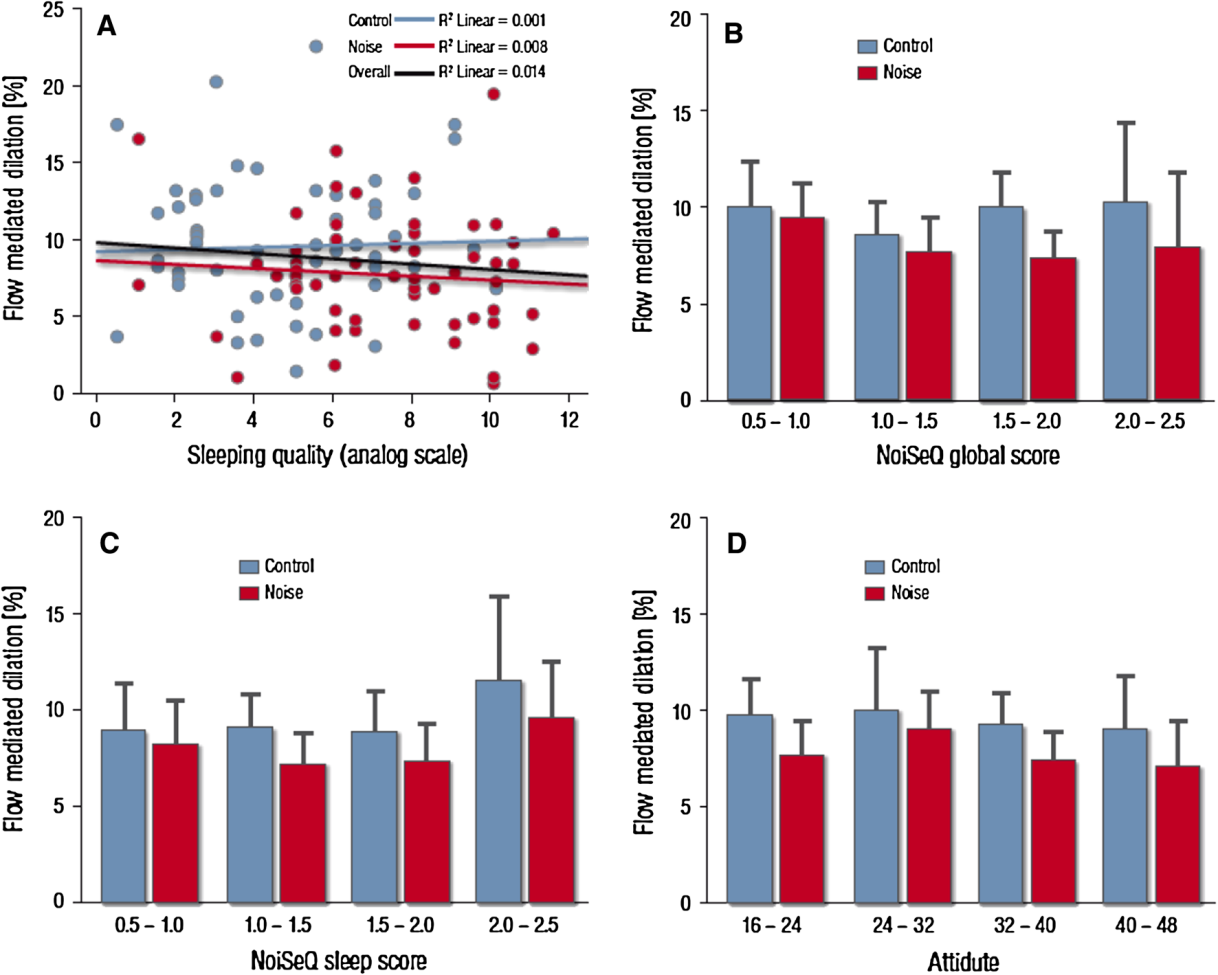
Influence of patient factors on flow-mediated dilation (FMD).** a** Subjective sleep quality (in cm as measured in the source data, higher values correspond to worse sleep quality; cumulative transformed data on a 0–10 scale are reported in Table 2) does not exhibit a significant correlation with FMD values.** b** Global noise sensitivity and** c** sleep related noise sensitivity as assessed by the Dortmund Noise Sensitivity Questionnaire (NoiSeQ) do not modify the effect of noise exposure on endothelial function (FMD).** d** Likewise patient attitude towards air traffic and aircraft noise does not predict the effect of noise simulation on the primary endpoint. Data are mean ±2 standard errors. Categories including* n* < 5 are not presented

In the linear mixed models, the response of participants’ endothelial function was not associated with overall noise sensitivity (NoiSeQ), sleep related noise sensitivity or attitude towards aircraft noise **(**Fig. 2).

Although subjective sleep quality was markedly impaired by the noise simulation, sleep quality (and sleep quality impairment) did not predict the blunting in endothelial responses on an individual level (Fig. 2).

### Secondary outcomes

**Fig. 3:**
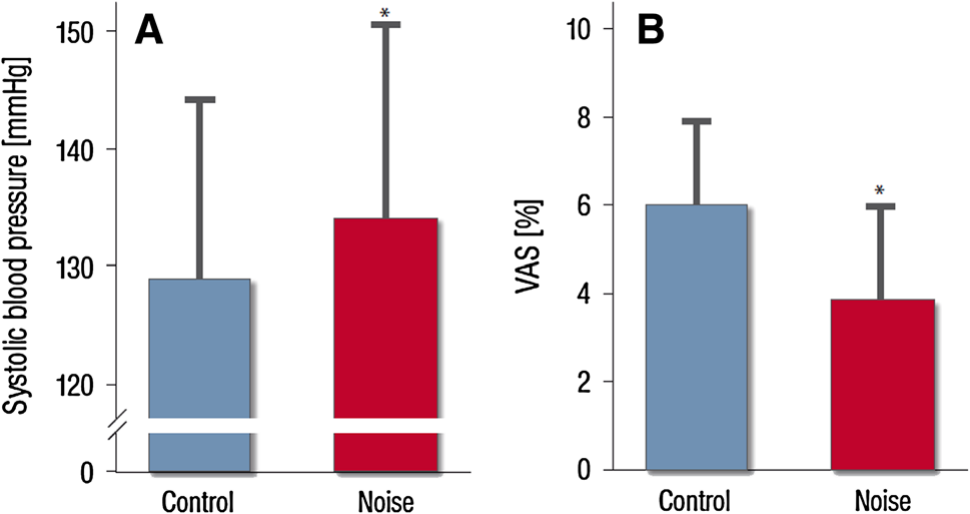
Effects of nighttime aircraft noise on systolic blood pressure **a** and sleep quality as expressed by the visual analog scale (VAS) **b**. Data are mean ± SD in 60 patients. Significance levels are *p = 0.03 (A) and *p < 0.001 **b** respectively adjusted for gender, age, night sequence, PSQI, overall noise sensitivity (NoiSeQ), sleep related noise sensitivity, attitude towards aircraft noise, and the results of the Morning Evening Questionnaire

Continuously measured systolic blood pressure increased from 129.5 ± 16.5 to 133.6 ± 17.9 mmHg (*p* = 0.030) (Fig. 3a).

Heart rate was not different between control and noise nights (60.7 ± 7.9 vs. 61.2 ± 7.9; *p* = 0.320; Table 2). With respect to the index of heart rate accelerations per time interval, there was a trend towards higher heart rate accelerations during noise exposure (8.9 ± 15.3 vs.13.5 ± 25.5; *p* = 0.059). The pNN50 index of heart rate variability did not change significantly.

The mean pulse transit time did not show any change after noise exposure (*p* = 0.450). Likewise, there was no evidence for a difference in neurohormonal parameters (adrenaline) and inflammatory markers (CRP, neutrophil count, IL-6) (Table 2).

Subjective sleep quality on a visual analog scale was markedly reduced by simulated aircraft noise from 5.8 ± 2.0 to 3.7 ± 2.2 (*p* < 0.001) (Fig. 3b).

Coagulation measures from a subset of study participants (*n* = 19) demonstrated just with respect Factor XII activity a significant reduction after noise exposure (96.4 ± 18.6 % vs. 89.4 ± 16.2 %; *p* = 0.004). All other coagulation factors remained unchanged (Table 3).

**Table 3:** Effects of nighttime noise on the activity of coagulation parameters

	Unit	Control	Noise	*p* value
F V	% activity	127.90 ± 16.10	125.01 ± 13.36	0.418
F VII	% activity	119.77 ± 19.56	117.38 ± 19.82	0.364
F VIII	% activity	122.16 ± 25.52	118.67 ± 26.12	0.527
F IX	% activity	120.60 ± 17.03	116.96 ± 13.15	0.188
F X	% activity	103.40 ± 14.18	100.43 ± 12.58	0.076
F XI	% activity	97.31 ± 17.12	102.96 ± 29.43	0.454
F XII	% activity	96.39 ± 18.49	89.39 ± 16.19	**0.004**
vWF	% activity	141.45 ± 40.02	128.94 ± 45.07	0.053
D-dimer	ng/ml	482.68 ± 827.18	544.68 ± 863.85	0.251

Bold value indicate statistically significance at the 5 % level

*vWF* von willebrand factor

## Discussion

The present data demonstrate for the first time that nighttime aircraft noise markedly attenuates endothelium-dependent vasodilation in patients with established and/or at high risk for CAD. The magnitude of this effect was such that the study was terminated early as the predefined stopping criteria were fulfilled at 60 % of the initially planned recruitment. An increase in blood pressure and a marked decrease in sleep quality were also observed in response to aircraft noise. Collectively, this evidence may concur to explain the reported association between nighttime aircraft noise and arterial hypertension, myocardial infarction and stroke.

### Aircraft noise and cardiovascular disease: evidence from epidemiological studies

A growing body of evidence documents that, beyond causing annoyance, aircraft noise should be considered a true cardiovascular risk factor (for review [5]).

In particular, recently published studies clearly substantiate the cardiovascular side effects of aircraft noise. In a multi-airport retrospective study in more than 6 Million people aged >65 years residing near airports, Correia et al. [11] reported a 3.5 % higher admission rate for cardiovascular disease such as ischemic coronary artery disease, cerebrovascular disease and heart failure for each 10 dB(A) increase in noise.

Another study in 3.6 million residents living close to Heathrow airport revealed that aircraft noise increased hospital admissions in a significant linear trend with increased risk with higher levels of both daytime and nighttime aircraft noise [12]. When areas experiencing the highest levels of daytime aircraft noise were compared with those experiencing the lowest levels (>63 dB vs. ≤51 dB), the relative risk of hospital admissions for stroke was 1.24 for coronary heart disease, 1.21 for cardiovascular disease and it remained 1.14 after adjustment for age, sex, ethnicity, deprivation, and a smoking proxy (lung cancer mortality). The authors concluded that high levels of aircraft noise were associated with increased risks of stroke, coronary heart disease and cardiovascular disease for both hospital admissions and mortality in areas near Heathrow airport in London.

Likewise, Floud et al. [13] reported a significant association between nighttime average aircraft noise and the endpoint ‘heart disease and stroke’ in the Hypertension and Environmental Noise near Airports (HYENA) study in 4,712 participants (276 cases) who lived near airports in six European countries (UK, Germany, Netherlands, Sweden, Greece, Italy). The result was not changed after adjustment for socio-demographic confounders for participants who had lived in the same place for ≥20 years. The authors concluded that exposure to aircraft noise over many years may increase risks of heart disease and stroke.

### Effects of simulated noise on vascular function

More recently, we provided some insight into potential mechanisms leading to vascular dysfunction and subsequently cardiovascular disease in response to nighttime aircraft noise. In a field study, we showed that nighttime aircraft noise simulation caused endothelial dysfunction and tended to increase blood pressure [6]. This pathophysiological study demonstrated the existence of a dose–response relationship between noise and endothelial function impairment, and the mechanism of noise-induced endothelial dysfunction was demonstrated to be linked with increased oxidative stress within the vasculature, because endothelial dysfunction was partly corrected by the antioxidant vitamin C. We also observed a priming effect, i.e. the impact of noise was larger when subjects had already been exposed to noise, suggesting that the vasculature is rather sensitized than preconditioned. The changes were paralleled and/or caused by increases in circulating adrenaline levels and were strongly associated with impaired sleep quality.

The present studies provide several important new information concerning cardiovascular risk and nighttime aircraft noise.

1. For the first time we demonstrate that nighttime noise substantially reduces FMD in patients with -or at risk for- coronary artery disease despite optimal, guideline conform concomitant cardiovascular medication including ACE-Inhibitors, AT-1 receptor blocker, statins and antiplatelet agents.

Importantly, endothelial dysfunction of forearm vessels has been shown to correlate well with endothelial dysfunction (and with the presence of atherosclerosis) in coronary vessels [14] and it has been shown to be associated with future cardiovascular events in patients with coronary and peripheral artery disease, heart failure, arterial hypertension and stroke (for review [15]).

2. In addition, there was evidence that nighttime aircraft noise simulation increased systolic blood pressure during the night by approximately 4 mmHg in patients with CAD. The observed 4 mmHg increase in systolic blood pressure is considered clinically relevant if sustained in the long-term, since recent studies have demonstrated that every 1 mmHg increase in systolic blood pressure in elderly with isolated systolic hypertension is associated with a 1 % increase new onset heart failure [16].

In the present studies, the increase in blood pressure was not associated with a change in plasma adrenaline concentration, possibly due to the background therapy with beta receptor-blocker or ACE/inhibitors and AT1 receptor blockers, substances that are known to have substantial inhibitory actions on the activity of the adrenergic and the renin angiotensin system [17, 18].

3. These data also demonstrate for the first time that detrimental vascular consequences in response to nighttime aircraft noise occur independently of the conscious perception of noise and the subject´s cognitive awareness (Fig. 2).

4. With regard to influence of noise on coagulation we detected a notable reduction in the level of Coagulation Factor XII (Hageman-Factor). While the role of factor XII in vivo remains controversial, associations of mildly reduced levels with increased coronary risk have been reported [19, 20].

The FMD measured in our patient group with cardiovascular disease was just slightly reduced compared to the FMD measured in healthy subjects [6], which may reflect optimal medical therapy.

It is important to note that our previous study clearly demonstrated that vessels are getting sensitized to noise-induced vascular damage rather than getting habituated since the blunting in FMD was in particular evident, when subjects were exposed first to 30 and then to 60 night noise events [6]. With the present studies we can already demonstrate a highly significant reduction in FMD in patients with CAD or being at high risk for CAD being exposed to 60 night noise events alone. Since patients living close to airports experience numerous noise events it seems likely that the degree of deterioration of endothelial function measured in the present study underestimates strongly the vascular damage induced by nighttime aircraft noise in the real world.

As in the previous study, noise exposure had a negative influence on subjective sleep quality of study participants. This finding may be regarded as clinically important, since self reported sleep quality is closely related to vascular calcification [21] and endothelial function [22]. Reduced sleep quality likely increases annoyance, which itself is associated with a higher probability of developing hypertension due to noise [23]. Recent evidence suggests a strong link between sleep duration and cardiovascular risk factors [24], which may explain increased extent of coronary artery disease lesions in persons with daytime sleepiness [25] due to insufficient sleep quality. The blunting in endothelial function and the increase in blood pressure were in our study not associated with impaired sleep quality on a patient level. These two adverse effects were also not prevented by guideline conform cardiovascular therapy.

In the current study, we could not detect changes in measures of immune function like cortisol levels, CRP, IL-6 or neutrophil count.

## Limitations of the study

The protocol was designed as a field study. While this limits the confounding influence of environment and equipment, thus creating ecologically valid conditions, ambient conditions, background sound levels and external stimuli could not be controlled for. Further, as only short-term effects of an environmental exposure were investigated here, conclusions on the long-term sequelae of such an exposure cannot be drawn from the present results.

FMD was chosen as surrogate endpoint for several reasons: as a functional measure, it is ideally suited to quantify functional changes of the vasculature to short-term exposure to noxious stimuli and it has shown to be predictive for future cardiovascular events in patients with hypertension, coronary and peripheral artery disease and stroke. FMD of forearm conductance vessels has also been demonstrated in a multicenter trial to have a high reproducibility [7]. In addition, sleep disturbances have just recently been demonstrated to adversely affect FMD [26].

The question whether a threshold of sound pressure levels beyond which the negative effects of noise appear, and what the best indicator of noise is, is often raised. Admittedly, our study setup cannot provide a definite answer to that. Nevertheless, there is now considerable evidence that a mean sound level of about 47 dB(A) such as that achieved in the present study consistently leads to moderate impairment of endothelial function in healthy controls [6] and to a substantial impairment of FMD patients with established or increased risk for coronary artery disease respectively (present study). Future studies will address the question whether mean or peak sound pressure levels are the important determinants leading to vascular dysfunction.

## Summary and clinical implications

The presented results demonstrate that nocturnal aircraft noise exposure causes severe endothelial dysfunction, raises systolic blood pressure in patients at high risk for cardiovascular events despite guideline oriented medical therapy. Taking into account the prognostic importance of endothelial function in patients with CAD, it is reasonable to conclude that nighttime aircraft noise-induced deterioration of vascular function may contribute at least in part to the observed increased incidence of arterial hypertension, MI and stroke in nighttime aircraft noise exposed people.

The present studies also stress the fact that more noise effect research has to be implemented and intensified. Important topics for the future may be to determine to what extent nighttime noise is able to modify the amount of circulating progenitor cells [27], whether noise-related stress is able to induce coronary vasomotor abnormalities [28] or to induce changes in biomarker such as nt-proBNP, which has been shown to have prognostic value for risk stratification in primary care [29].

Importantly, when decisions concerning the location and/or activity volume of airports are taken, the impact of aircraft noise on the health of the surrounding population should be considered. While many of the traditional risk factors (e.g. smoking, cholesterol levels, diabetes and hypertension) can be modified by the patients´ behavior and attitudes, nighttime aircraft noise can be considered as the only risk factor, which can only be changed by politicians and not by the patient himself.

## References

[1] Basner M, Babisch W, Davis A, Brink M, Clark C, Janssen S, Stansfeld S (2014) Auditory and non-auditory effects of noise on health. Lancet 383(9925):1325–1332 10.1016/S0140-6736(13)61613-X PMC3988259 24183105

[2] Jarup L, Babisch W, Houthuijs D, Pershagen G, Katsouyanni K, Cadum E, Dudley ML, Savigny P, Seiffert I, Swart W, Breugelmans O, Bluhm G, Selander J, Haralabidis A, Dimakopoulou K, Sourtzi P, Velonakis M, Vigna-Taglianti F (2008). Hypertension and exposure to noise near airports: the HYENA study. Environ Health Perspect.

[3] van Kempen E, van Kamp I, Fischer P, Davies H, Houthuijs D, Stellato R, Clark C, Stansfeld S (2006). Noise exposure and children’s blood pressure and heart rate: the RANCH project. Occup Environ Med.

[4] Greiser E, Greiser C, Jahnsen K (2007). Night-time aircraft noise increases the prescription for antihypertensive and cardiovascular drugs irrespective of social class-the Cologne-Bonn Airport study. J Public Health.

[5] Munzel T, Gori T, Babisch W, Basner M (2014) Cardiovascular effects of environmental noise exposure. Eur Heart J 35(13):829–836 10.1093/eurheartj/ehu030 PMC3971384 24616334

[6] Schmidt FP, Basner M, Kroger G, Weck S, Schnorbus B, Muttray A, Sariyar M, Binder H, Gori T, Warnholtz A, Munzel T (2013) Effect of nighttime aircraft noise exposure on endothelial function and stress hormone release in healthy adults. Eur Heart J 10.1093/eurheartj/eht269 PMC3844151 23821397

[7] Charakida M, Deanfield JE (2013). Nighttime aircraft noise exposure: flying towards arterial disease. Eur Heart J.

[8] Schnorbus B, Schiewe R, Ostad MA, Medler C, Wachtlin D, Wenzel P, Daiber A, Munzel T, Warnholtz A (2010). Effects of pentaerythritol tetranitrate on endothelial function in coronary artery disease: results of the PENTA study. Clin Res Cardiol.

[9] Pocock SJ (2005). When (not) to stop a clinical trial for benefit. JAMA.

[10] Schulz KF, Grimes DA (2005). Multiplicity in randomised trials II: subgroup and interim analyses. Lancet.

[11] Correia A, Peters JL, Levy JI, Melly S, Dominici F (2013). Residential exposure to aircraft noise and hospital admissions for cardiovascular diseases: multi-airport retrospective study. Br Med J.

[12] Hansell AL, Blangiardo M, Fortunato L, Floud S, de Hoogh K, Fecht D, Ghosh RE, Laszlo HE, Pearson C, Beale L, Beevers S, Gulliver J, Best N, Richardson S, Elliott P (2013). Aircraft noise and cardiovascular disease near Heathrow airport in London: small area study. BMJ.

[13] Floud S, Blangiardo M, Clark C, de Hoogh K, Babisch W, Houthuijs D, Swart W, Pershagen G, Katsouyanni K, Velonakis M, Vigna-Taglianti F, Cadum E, Hansell AL (2013). Exposure to aircraft and road traffic noise and associations with heart disease and stroke in six European countries: a cross-sectional study. Environ Health.

[14] Anderson TJ, Uehata A, Gerhard MD, Meredith IT, Knab S, Delagrange D, Lieberman EH, Ganz P, Creager MA, Yeung AC (1995). Close relation of endothelial function in the human coronary and peripheral circulations. J Am Coll Cardiol.

[15] Lerman A, Zeiher AM (2005). Endothelial function: cardiac events. Circulation.

[16] Ekundayo OJ, Allman RM, Sanders PW, Aban I, Love TE, Arnett D, Ahmed A (2009). Isolated systolic hypertension and incident heart failure in older adults: a propensity-matched study. Hypertension.

[17] Sigurdsson A, Swedberg K (1995). Neurohormonal activation and congestive heart failure: today’s experience with ACE inhibitors and rationale for their use. Eur Heart J.

[18] Remme WJ (1998). The sympathetic nervous system and ischaemic heart disease. Eur Heart J.

[19] Bach J, Endler G, Winkelmann BR, Boehm BO, Maerz W, Mannhalter C, Hellstern P (2008). Coagulation factor XII (FXII) activity, activated FXII, distribution of FXII C46T gene polymorphism and coronary risk. J Thromb Haemost.

[20] Lessiani G, Falco A, Nicolucci E, Rolandi G, Davi G (2009). Deep venous thrombosis and previous myocardial infarction in mild factor XII deficiency: a risk factor for both venous and arterial thrombosis. J Thromb Thrombolysis.

[21] Matthews KA, Everson-Rose SA, Kravitz HM, Lee L, Janssen I, Sutton-Tyrrell K (2013). Do reports of sleep disturbance relate to coronary and aortic calcification in healthy middle-aged women?: study of women’s health across the nation. Sleep Med.

[22] Behl M, Bliwise D, Veledar E, Cunningham L, Vazquez J, Brigham K, Quyyumi A (2014) Vascular endothelial function and self-reported sleep. Am J Med Sci 347(6):425–428 10.1097/MAJ.0b013e31829bc950 PMC3852200 23842206

[23] Babisch W, Pershagen G, Selander J, Houthuijs D, Breugelmans O, Cadum E, Vigna-Taglianti F, Katsouyanni K, Haralabidis AS, Dimakopoulou K, Sourtzi P, Floud S, Hansell AL (2013). Noise annoyance–a modifier of the association between noise level and cardiovascular health?. Sci Total Environ.

[24] Grandner MA, Chakravorty S, Perlis ML, Oliver L, Gurubhagavatula I (2014). Habitual sleep duration associated with self-reported and objectively determined cardiometabolic risk factors. Sleep Med.

[25] Lee CH, Ng WY, Hau W, Ho HH, Tai BC, Chan MY, Richards AM, Tan HC (2013). Excessive daytime sleepiness is associated with longer culprit lesion and adverse outcomes in patients with coronary artery disease. J Clin Sleep Med.

[26] Cooper DC, Ziegler MG, Milic MS, Ancoli-Israel S, Mills PJ, Loredo JS, Von Kanel R, Dimsdale JE (2014). Endothelial function and sleep: associations of flow-mediated dilation with perceived sleep quality and rapid eye movement (REM) sleep. J Sleep Res.

[27] Hoefer IE, Sels JW, Jukema JW, Bergheanu S, Biessen E, McClellan E, Daemen M, Doevendans P, de Groot P, Hillaert M, Horsman S, Ilhan M, Kuiper J, Pijls N, Redekop K, van der Spek P, Stubbs A, van de Veer E, Waltenberger J, van Zonneveld AJ, Pasterkamp G (2013). Circulating cells as predictors of secondary manifestations of cardiovascular disease: design of the circulating cells study. Clin Res Cardiol.

[28] Ong P, Athanasiadis A, Perne A, Mahrholdt H, Schaufele T, Hill S, Sechtem U (2014). Coronary vasomotor abnormalities in patients with stable angina after successful stent implantation but without in-stent restenosis. Clin Res Cardiol.

[29] Leistner DM, Klotsche J, Pieper L, Palm S, Stalla GK, Lehnert H, Silber S, Marz W, Wittchen HU, Zeiher AM (2013). Prognostic value of NT-pro-BNP and hs-CRP for risk stratification in primary care: results from the population-based DETECT study. Clin Res Cardiol.

